# A patient with sudden pulmonary embolism and stroke after total hysterectomy and bilateral salpingo-oophorectomy was diagnosed with patent foramen ovale: case report and review

**DOI:** 10.3389/fcvm.2025.1666061

**Published:** 2025-08-29

**Authors:** Lijun Zhao, Boyuan Wang, Bei Zhao, Ye Tian, Rui Zhai, Mei Hu

**Affiliations:** ^1^ Department of Critical Care Medicine, The Ninth Medical Center of Chinese People’s Liberation Army General Hospital, Beijing, China; ^2^ Department of Cardiology, The Ninth Medical Center of Chinese People’s Liberation Army General Hospital, Beijing, China

**Keywords:** patent foramen ovale, pulmonary embolism, cerebral infarction, paradoxical embolism, status post total hysterectomy with bilateral salpingo-oophorectomy, case report

## Abstract

A 75-year-old woman was admitted for the treatment of uterine fibroids and underwent laparoscopic total hysterectomy with bilateral salpingo-oophorectomy under general anesthesia. On the first postoperative day, the patient experienced sudden convulsion followed by loss of consciousness while transitioning from a seated to standing position. Subsequent examinations revealed pulmonary embolism and partial thrombosis in the muscular veins of the right lower leg. Anticoagulant therapy was immediately initiated. On postoperative day 3, early morning, the patient was found to be agitated with a positive Babinski sign on the right side. A CT scan of the brain revealed a cerebral infarction. Following the sequential occurrence of pulmonary embolism and cerebral infarction, paradoxical embolism drew the physician's attention. After discussion, the cause was attributed to either an arteriovenous fistula or a patent foramen ovale. Subsequent transesophageal echocardiography (TEE) and bubble study confirmed a patent foramen ovale (PFO) in the patient. This case highlights the critical importance of proactively searching for underlying etiologies when faced with such abnormal clinical presentations.

## Introduction

Patent foramen ovale (PFO) refers to the failure of the normal fetal communication between the left and right atria (the foramen ovale) to close after birth (1). PFO is a common condition, occurring in approximately 25% of the population. Diagnostic options include transthoracic echocardiography (TTE), transesophageal echocardiography (TEE), and transcranial Doppler ultrasonography (TCD) (2). A meta-analysis demonstrated an association between PFO and an elevated risk of perioperative stroke during non-cardiac surgery (3). The potential mechanisms of stroke include paradoxical embolism from venous thrombi traversing the PFO, *in situ* thrombosis within the PFO, and atrial arrhythmias resulting from electrical conduction disturbances (4).

Globally, stroke is the second leading cause of death, and the disease is clinically characterised by sudden-onset neurological deficits (5). Approximately 25%–40% of ischemic strokes occur without an identifiable cause and are classified as cryptogenic stroke (6). In 1988, P Lechat et al. first reported that the presence of a PFO increases the risk of ischemic stroke and is a common cause of cryptogenic stroke (7). Thrombi derived from the venous circulation embolize into the systemic circulation through right-to-left shunting permitted by the PFO, leading to stroke (8). A prospective cohort study demonstrated that among patients with symptomatic pulmonary embolism, the patient with PFO had a higher incidence of recent stroke compared to those without PFO (9). This finding further supports paradoxical embolism as a significant mechanism of ischemic stroke in PFO patients. The study reported a case of a uterine fibroid patient who developed pulmonary embolism followed by cerebral stroke after undergoing total hysterectomy with bilateral adnexectomy. This clinical presentation promptly prompted clinicians to suspect paradoxical embolism, ultimately leading to the diagnosis of a PFO.

## Case presentation

A 75-year-old female patient was admitted to the Department of Gynecology due to uterine fibroids (measuring 6.2 × 4.1 cm). She reported no recent symptoms such as headache, dizziness, or fatigue. The patient underwent left thyroid cystectomy 30 years ago and denied other significant medical history.

**Figure 1:**
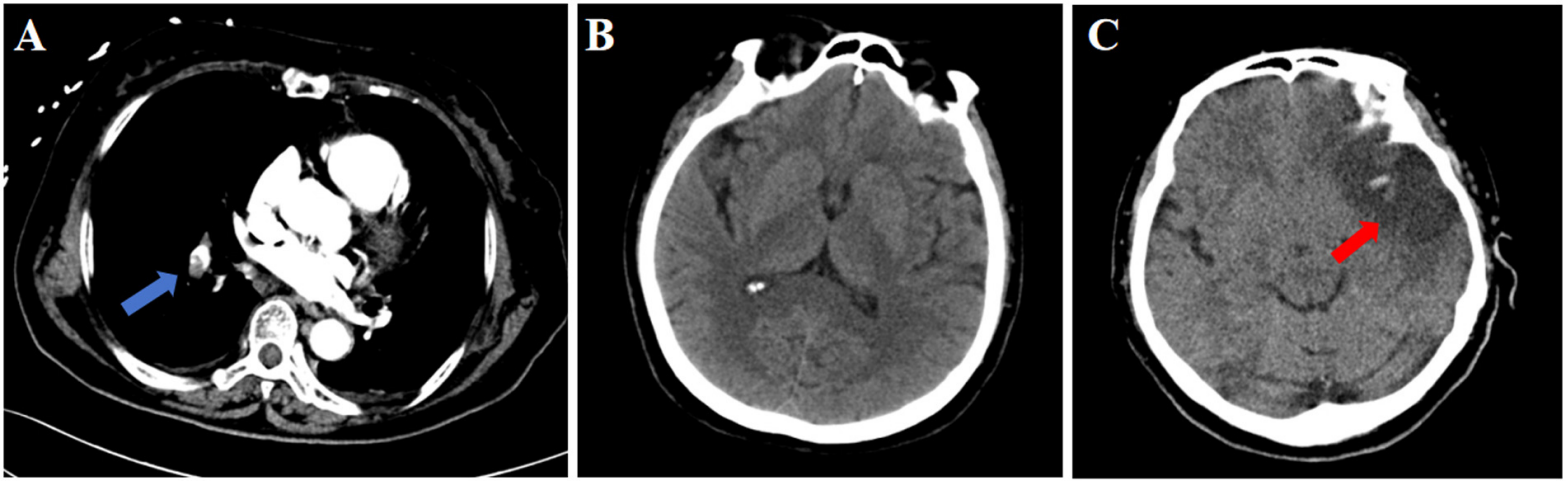
CT findings of the patient. **(A)** Pulmonary CT on postoperative day 1; **(B)**. Cranial CT on postoperative day 1; **(C)**. Cranial CT on postoperative day 3. Blue arrow: pulmonary embolism; Red arrow: stroke.

The patient underwent laparoscopic total hysterectomy with bilateral salpingo-oophorectomy under general anesthesia after admission. Intraoperative vital signs remained stable, and the procedure was completed uneventfully. On the first postoperative day, the patient experienced sudden convulsion and loss of consciousness while transitioning from a sitting to standing position, becoming unresponsive to verbal stimuli. Immediate physical examination revealed: Blood pressure 92/64 mmHg, heart rate 72 bpm, oxygen saturation 67%. Bilateral pupils were equal and round with preserved light reflex. Clear breath sounds were heard in both lung fields without rales or rhonchi. Arterial blood gas analysis revealed: pH 7.27, PO₂ 59.0 mmHg, PCO_2_ 35 mmHg, Glucose 10.6 mmol/L, Lactate 3.5 mmol/L, Base Excess (BEb) −9.9 mmol/L. Immediate endotracheal intubation was performed with initiation of mechanical ventilation. Enhanced pulmonary CT angiography revealed pulmonary embolism (Figure 1A), while brain CT showed no significant abnormalities (Figure 1B). Transthoracic echocardiography (TTE) demonstrated trivial tricuspid regurgitation and impaired left ventricular relaxation. Lower extremity venous doppler ultrasound demonstrated partial muscular vein thrombosis in the right calf. Low molecular weight heparin calcium (0.6 ml Q12 h) was promptly initiated for anticoagulation. Subsequently, intermittent pneumatic compression (IPC) was initiated for the patient. Due to critical condition, the patient was immediately transferred to the Intensive Care Unit (ICU).

**Figure 2:**
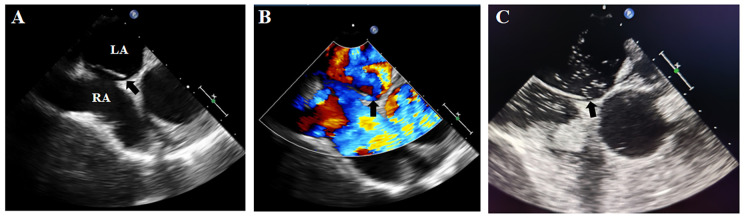
Transesophageal echocardiographic findings. **(A,B)** Demonstrated a significant PFO with right-to-left shunting; **(C)**: strongly positive bubble study. Black arrow: PFO. LA, left atrium; RA, right atrium.

On postoperative day 3, early morning, the patient developed agitation and right-sided limb weakness with a positive Babinski sign on the right. Further brain CT demonstrated hypodense lesions indicating large-territory cerebral infarction in the left hemisphere (Figure 1C). Given the sequential occurrence of pulmonary embolism and cerebral infarction, the treating physicians clinically suspected a pathological right-to-left shunt pathway, such as arteriovenous fistula or PFO. TEE and bubble study was performed, revealing: Patent foramen ovale with significant right-to-left shunt (Figures 2A,B). Strongly positive bubble study (Figure 2C). The final diagnosis was PFO, pulmonary embolism and cerebral infarction.

The patient was successfully stabilized from the critical condition and discharged from the ICU. We have advised the patient to consult an interventional cardiologist to evaluate the feasibility of PFO closure.

## Case review

To further enhance our comprehension of this disease category, we conducted a systematic review of case reports spanning the past two decades (January 2005–June 2025). We performed a PubMed search spanning all fields from 2005 to present using the query “(patent foramen ovale) AND (Pulmonary Embolism) AND (stroke)”, yielding 148 initial results. To ensure quality assurance, only Science Citation Index-indexed case reports were considered. Given the predominance of publications describing PFO patients with either stroke or pulmonary embolism alone, we manually curated articles documenting concurrent or sequential pulmonary embolism and stroke in PFO patients (≥18 years), ultimately identifying 32 qualifying cases (Supplementary Figure S1). Among these cases, 18 were male and 14 were female. Thrombotic events manifested abruptly in the majority of patients, with only 4 cases having recent surgical procedures prior to onset. The sequence of thrombotic events was documented as follows: pulmonary embolism preceding cerebral stroke in 9 patients, cerebral stroke detected prior to pulmonary embolism in 4 patients, and 19 patients with simultaneous detection of pulmonary embolism and stroke or unrecorded order of detection. Although the median age of patients with first-diagnosed cryptogenic stroke associated with PFO is around 45 years, showing regional trends toward younger onset (10), our analysis of reported cases identified 19 patients aged over 60 years. This requires heightened vigilance as PFO-related stroke in older patients should not be overlooked; PFO closure was confirmed in 12 cases. While studies indicate that PFO closure reduces stroke recurrence rates in patients with high-risk PFO features, the reported mean patient age was only 51.8 years (11). A multicenter study on PFO closure in elderly patients demonstrated that compared to the control group's recurrent cerebrovascular events and stroke rate of 1.21%, the intervention group exhibited a reduced incidence of 0.55%; however, it must be emphasized that diabetes, aspirin use, and advanced age are factors associated with increased risk of adverse clinical outcomes; cautious consideration is therefore required in clinical decision-making regarding PFO closure for this specific population (12).

**Table 1:** Case review details.

No.	Authors	Date of publication	Patient's age	Pre-onset intervention	Symptom	Embolism site	Treatment
1	Bracey et al. (13)	2006	29	NA	Severe chest pain and dyspnea at rest, with right-sided limb weakness, loss of consciousness, and respiratory arrest	Lung, brain, deep vein of the left lower extremity, right atrium, right ventricle, left atrium, and aortic arch extending to the left common carotid artery	NA
2	Iwanaga et al. (14)	2007	84	NA	Coma, tetraplegia	Brain, lung, lower limb deep venous	NA
3	Shibazaki et al. (15)	2007	79	NA	Sudden onset of consciousness disturbance, left central facial palsy and left hemiplegia were evident	Lung, brain	NA
4	Pavesi et al. (16)	2008	65	Air travel	Worsening dyspnea, chest pain	Lung, brain	NA
5	Seetharaman et al. (17)	2011	62	NA	Left-sided weakness, left-sided hemianopia, hemineglect, hemiplegia and hemianaesthesia, and a swollen left calf	Brain, lung	PFO closure procedure
6	Chow et al. (18)	2012	35	NA	Collapsed, loss of consciousness, unable to speak	Brain, lung	NA
7	Lewis et al. (19)	2012	66	NA	Speech loss, right-sided weakness	Brain, lung	PFO closure procedure
8	Kumar et al. (20)	2013	32	NA	Right-sided hemiparesis	Brain, lung, right popliteal vein, common iliac vein	NA
9	Omar et al. (21)	2013	69	Right total hip replacement	Sudden dyspnoea, wheezing and confusion	Brain, lung	NA
10	Cameron et al. (22)	2015	53	NA	Left gaze preference, dysarthria, and flaccid right hemiplegia	Brain, lung	NA
11	Miriyala et al. (23)	2016	72	NA	Left-sided weakness, confusion, and altered speech	Brain, lung	NA
12	Urja et al. (24)	2017	73	NA	Slurred speech, near-syncope, dizziness, and progressive left-sided limb weakness	Lung, brain	NA
13	Barros-Gomes et al. (25)	2018	68	NA	Facial deviation, right upper extremity weakness, and aphasia	Bilateral lower extremities, brain, lung	NA
14	Konala et al. (26)	2019	82	NA	Lightheadedness, mild dyspnea, and sudden syncope.	Lung, brain	NA
15	De Sousa Bispo et al. (27)	2019	80	NA	Positional dyspnoea that develops upon sitting up, accompanied with refractory hypoxaemia	Brain, lung	PFO closure procedure
16	Lak et al. (28)	2020	68	NA	Acute visual impairment manifesting as partial hemianopsia	Brain, lung, superior mesenteric artery	PFO closure procedure
17	Jena et al. (29)	2020	64	NA	Confusion, slurred speech, right-sided weakness with pronator drift, right facial droop, and preserved forehead movement	Brain, lung	NA
18	Gunn et al. (30)	2020	66	NA	Acute respiratory failure	Lung, brain	PFO closure procedure
19	Suenaga et al. (31)	2021	55	NA	Syncope resulting in a fall	Lung, brain	NA
20	Kass et al. (32)	2021	67	Prostatic surgery	Hematuria, urinary retention, and hypoxemia with dysarthria	Lung, brain, bladder	PFO closure procedure
21	Galtes et al. (33)	2021	42	NA	Agitation, confusion accompanied by generalized weakness, aphasia, and right-sided hemiplegia.	Brain, lung	PFO closure procedure
22	Jayalakshmi et al. (34)	2021	52	NA	Sudden left hemiparesis	Brain, lung	NA
23	Takemoto et al. (35)	2021	77	NA	Dyspnea followed by cardiac arrest	Lung, brain	NA
24	Dattani et al. (36)	2022	55	NA	Sudden dyspnoea, left limb weakness	Lung, brain	PFO closure procedure
25	He et al. (37)	2022	42	NA	Left chest pain, shortness of breath and lower extremity edema	Brain, lung, left common femoral vein, femoral vein, popliteal vein, inferior vena cava, bilateral external iliac veins, and bilateral internal iliac veins	PFO closure procedure
26	Uecker et al. (38)	2023	57	NA	Dyspnoea	Lung, brain	PFO closure procedure
27	Zaussinger et al. (39)	2023	36	Cosmetic breast augmentation-mastopexy	Frequent generalized seizures	Lung, brain	NA
28	Alaboud Alkheder et al. (40)	2024	61	NA	Loss of consciousness, tachypnea accompanied by right-sided limb weakness and visual changes	brain, lung	NA
29	Tyler et al. (41)	2024	66	Orthotopic liver transplantation	Acute onset of shortness of breath, left-sided limb weakness, facial droop, and altered mental status	Brain, lung	PFO closure procedure
30	Ahmad et al. (42)	2024	50	NA	Chest pain and respiratory distress	Brain, lung	PFO closure procedure
31	Quasem et al. (43)	2025	33	A history of intravenous drug use	Infection, acute confusion	Lung, brain	NA
32	Monteiro et al. (44)	2025	86	NA	Acute onset global aphasia, right-sided hemiplegia, left conjugate gaze deviation, right homonymous hemianopsia with facial weakness	Brain, lung	NA

## Discussion

The incidence of postoperative venous thromboembolism (VTE), including pulmonary embolism, following laparoscopic hysterectomy is reported to be approximately 0.3% (45). In the case we presented, the patient underwent a laparoscopic total hysterectomy with bilateral salpingo-oophorectomy. On postoperative day 1, she developed a pulmonary embolism. A brain CT scan revealed no abnormalities. Additionally, TTE did not detect pulmonary hypertension in the patient. Based on the patient's medical history, the physicians initially suspected the pulmonary embolism was a postoperative complication. However, on the night of the second postoperative day, the patient became agitated and developed impaired motor function in the right limbs. A Babinski sign was positive on the right side. Suspecting an intracranial abnormality, the physicians performed a further brain CT scan, which confirmed the presence of a stroke. Based on human physiology, physicians noted that pulmonary embolism does not typically lead to stroke under normal circumstances. This paradoxical embolism prompted significant concern among the treating physicians, who suspected the presence of either an arteriovenous fistula or a PFO. The diagnosis of PFO was ultimately confirmed through TEE with a bubble study. We had hypothesized that pulmonary hypertension might facilitate paradoxical embolism; however, TTE showed no evidence of this phenomenon in the patient.

A notable feature of this case is that the patient developed cerebral infarction (stroke) shortly after pulmonary embolism in the postoperative period. This leading to the diagnosis of a PFO. Existing case reports have documented similar occurrences of paradoxical embolism due to a PFO in postoperative patients (32). However, acute sequential pulmonary embolism and cerebral infarction following total hysterectomy with bilateral salpingo-oophorectomy have not been documented. Current preventive strategies for postoperative thromboembolism—early ambulation, intermittent pneumatic compression, and indicated early anticoagulation—are relatively low-cost. A large-scale observational study revealed that preoperatively diagnosed patent PFO is associated with an increased risk of ischemic stroke within 30 days after surgery (46). A meta-analysis of PFO and perioperative stroke in non-cardiac surgery revealed that PFO presence correlates with heightened stroke risk in surgical patients (3). The study proposes that early PFO identification and preventive protocol development may potentially improve perioperative outcomes. Therefore, perioperative screening for PFO could be beneficial, but the high cost and low detection rate might make it impractical. Certainly, the impact of preoperative diagnosis of PFO and its corresponding treatment on stroke prevention still requires validation through large-scale randomized clinical trials. We hope our report will provide clinicians with valuable reference.

## Data Availability

The original contributions presented in the study are included in the article/Supplementary Material, further inquiries can be directed to the corresponding author.
