# Supplementary figures and images for: A patient with sudden pulmonary embolism and stroke after total hysterectomy and bilateral salpingo-oophorectomy was diagnosed with patent foramen ovale: case report and review
Source: Front Cardiovasc Med. 2025 Aug 29;12:1666061. doi: 10.3389/fcvm.2025.1666061 (PMC12426153; doi:10.3389/fcvm.2025.1666061)

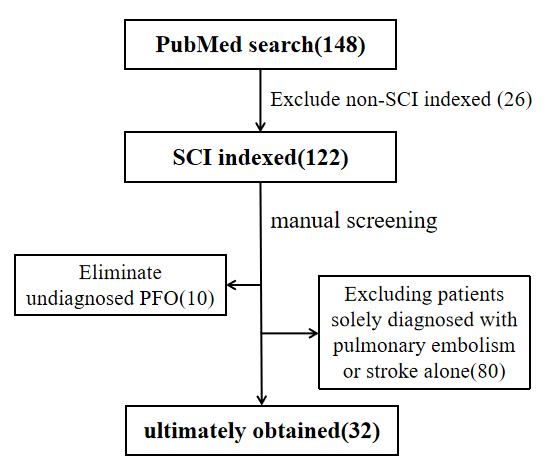

Supplement: Supplementary file 1 [file Image1.jpg]
